# Agalsidase alfa and agalsidase beta in the treatment of Fabry disease: does the dose really matter?

**DOI:** 10.1038/gim.2014.79

**Published:** 2014-07-10

**Authors:** Antonio Pisani, Eleonora Riccio, Massimo Sabbatini

**Affiliations:** 1 Section of Nephrology, Department of Public Health, Federico II University of Naples, Naples, Italy

Fabry disease (FD) is a multiorgan, X-linked lysosomal storage disorder that particularly affects the heart, the kidneys, and the cerebrovascular system.^1^ The treatment options for patients with FD include long-term enzyme replacement therapy (ERT) in addition to supportive management. Two recombinant enzyme formulations for the ERT of FD are available on the European market: agalsidase alfa (Replagal; Shire Human Genetic Therapies AB, Danderyd, Sweden) and agalsidase beta (Fabrazyme; Genzyme Corporation, Cambridge, MA).^1^ Numerous clinical trials, observational studies, and registry data have provided evidence about the safety and efficacy of ERT^2^; to date, however, there have been limited comparisons between the two agents, and no firm conclusion regarding their specific efficacy and safety can be made.

A viral contamination in the manufacturing process of Fabrazyme in June 2009 led to a global shortage of agalsidase beta. Recommendations to reduce the dosage of the drug were consequently published by the European Medicines Agency for patients receiving agalsidase beta; this obviously caused fear and concern among both patients and physicians. On the basis of an increased rate of serious adverse effects in patients administered reduced doses,^3^ a subsequent European Medicines Agency report suggested restarting treatment with full-dose agalsidase beta or shifting patients to recommended doses of agalsidase alfa.

Therefore, after a period of reduced dosage of agalsidase beta, many patients were switched to agalsidase alfa. This offered the unique opportunity to compare the two drugs, albeit indirectly, evaluating any clinical modifications or adverse events that occurred after the switch. In 2011, in a Dutch cohort of 35 patients with FD who continued agalsidase beta at reduced doses or were switched to agalsidase alfa after about 5 months of low-dose agalsidase beta, Smid et al.^4^ showed that renal function, left-ventricular mass, symptoms of pain, and incidence of clinical events were not significantly altered during the shortage; quality of life was minimally but significantly affected in females in two subscales of the 36-Item Short Form Health Survey; more important, an increase in lyso-Gb3, a marker of disease involvement, was observed in males after 1 year of therapy at either low doses of agalsidase beta or a full dose of agalsidase alfa.

One year later, Tsuboi and Yamamoto^5^ presented the results of an observational study involving 11 patients who switched from agalsidase beta (1 mg/kg every other week) to agalsidase alfa (0.2 mg/kg every other week): renal function, cardiac mass, and quality of life remained stable throughout the 12-month follow-up. Similarly, our group^6^ evaluated the effect of such a switch in 10 patients with FD (7 males, 3 females) who were previously treated with agalsidase beta for at least 48 months. The results showed that renal function, cardiac mass assessed by magnetic resonance imaging, symptoms of pain, and health status scores remained stable throughout the 24-month follow-up period. More recently, Weidemann et al.^7^ reported their experience during the agalsidase beta shortage that resulted in a change of treatment regimen in many patients. They assessed end-organ damage and clinical symptoms among 105 patients with FD who were previously treated with agalsidase beta (1.0 mg/kg every other week for ≥1 year) and who were arbitrarily assigned, on the basis of their symptoms, to continue their treatment regimen, to receive a reduced dose of agalsidase beta (0.3–0.5 mg/kg), or to be switched to the full dose of agalsidase alfa (0.2 mg/kg). No clinical event occurred after dose reduction or compound switch, as already observed by Tsuboi and Yamamoto^5^ and us. However, Weidemann et al.^7^ reported a significant deterioration of Fabry-related symptoms in both groups, a significant decline in glomerular filtration rate estimated using cystatin in the dose-reduction group and a significant increase in urinary albumin-to-creatinine ratio only in patients switched to agalsidase alfa. This result was stressed by Warnock and Mauer^8^ in a recent editorial emphasizing that the dose of the drug “matters” in FD treatment and suggesting that the full dosage of agalsidase alfa could be too low to guarantee results as effective as those of agalsidase beta.^8^ However, there are some considerations to be taken into account when considering all the studies dealing with the shift to agalsidase alfa: because of their observational nature, the unavoidable selection of patients, the short follow-up, and the low number of events observed after ERT introduction, the intrinsic limits of these studies do not allow final conclusions about the efficacy and safety of agalsidase alfa to be made.

Indeed, no renal biopsy was performed during the follow-up in shifted patients, able to demonstrate greater podocyte injury and/or new deposition of Gb3 in tubular cells.^1^ It was impossible to demonstrate the injury because renal biopsy was not performed. Moreover, the increased levels of lyso-Gb3 described by Smid et al.^4^ 1 year after the shift were observed in patients previously treated with a low dose of agalsidase beta for 6 months; if dose matters, such a finding could be ascribed to the reduced dose of agalsidase beta and not to agalsidase alfa.

The observed twofold increase in the albumin-to-creatinine ratio, described by Weidemann et al.^7^ after a 12-month treatment with agalsidase alfa, in the presence of a relatively stable cystatin-C-based glomerular filtration rate, could suggest a “true” worsening of renal function. However, the same patients had already shown a 2.7-fold increase in this parameter during the preceding year (i.e., while receiving the full dose of agalsidase beta). The characteristics and the clinical treatment of these patients may explain the progressive and continuous increase in albuminuria; in fact, it is interesting to note that these patients were less protected by renin–angiotensin system blockers, which were administered to only 24% of shifted patients compared with 58% of patients in the dose reduction group and 34% of patients receiving the full dose of agalsidase beta. Although such a difference was not significant, it is widely accepted that proteinuria does not respond solely to ERT,^1^ and renin–angiotensin system blockers represent a critical stabilizing factor of proteinuria.^1^ Therefore, a specific role for agalsidase alfa in worsening proteinuria should be reconsidered.

Finally, the significant increase in adverse events, such as gastrointestinal symptoms, pain attacks, or chronic pain, during agalsidase alfa treatment is difficult to interpret and quantify adequately. It is not possible to exclude that the anxiety caused by the drug shortage and by European Medicines Agency warnings led to the increased reporting of adverse events by patients and greater attention given to their diagnosis by physicians. This has probably overestimated the real incidence of these “subjective” symptoms. It is much more important to stress that, under agalsidase alfa treatment, “objective” targets, such as cardiac measures or neurologic involvement, were not affected, and the number of events remained stable despite the short observation period.

The recent data by Tsuboi and Yamamoto^9^ support the safety of switching from agalsidase beta to agalsidase alfa at the approved doses, without loss of efficacy on organ involvement over a long-term period. They reported data from 11 patients switched from agalsidase beta to agalsidase alfa during a prolonged follow-up; in fact, clinical data were collected for 5 years—2 years before and 3 years after the switch. Their results showed that renal function remained stable during the last 3 years and that the improvements in cardiac mass, recorded 12 months after switching to agalsidase alfa, were maintained throughout the follow-up. Moreover, there was no significant difference in pain severity and quality-of-life parameters evaluated before and after switching.

Our recent data from 10 patients with FD who were previously switched to agalsidase alfa further support these results. In fact, with the increased availability of agalsidase beta in the last quarter of 2012, five patients (three males) returned to full-dose agalsidase beta (1.0 mg/kg every other week) after a 30-month average treatment with agalsidase alfa, whereas the remaining five patients (four males) continued their ongoing therapy with agalsidase alfa (0.2 mg/kg). To date, the follow-up of these 10 patients averages 40 months after the first switch to agalsidase alfa. As in our previous study,^7^ we evaluated renal function, selected cardiac parameters, pain symptoms, and patient health status either at baseline (i.e., 20 months after the switch) and after 20 further months of continuous agalsidase alfa or 20 months after the switch back to agalsidase beta. There was no difference in age between the two groups (total mean, 43.5 ± 5.5 years) nor in the estimated glomerular filtration rate (total mean, 91.1 ± 14.9 mL/minute), and all patients had a described mutation expressing the classic FD phenotype with severe multiorgan involvement, which makes unlikely the hypothesis that they had a stable or a slowly progressing disease. Our data demonstrate that no clinical event occurred during the follow-up period in any group using the approved drug doses. Throughout the follow-up period, renal function remained stable in both groups, and no change was observed in median urinary protein-to-creatinine ratio nor in cardiac function assessed by left-ventricular ejection fraction and by changes in left-ventricular mass on cardiac magnetic resonance imaging, as compared with values before the shift. Finally, symptoms of pain and health status scores did not worsen during the follow-up. Agalsidase alfa was well tolerated throughout the observation period, and no clinical problem occurred after the reintroduction of agalsidase beta in patients who switched back. Despite the exiguous number of patients involved in this observation, and considering that 80% of the patients treated with this drug were males, who are more prone to disease progression, these data offer further information about the safety and efficacy of agalsidase alfa. A recent report showed two cases of significant clinical improvement of severe adverse events on an approved/reduced dose of agalsidase beta after the switch to agalsidase alfa.^10^ obviously, we need to get further information from all the centers involved in the switch policy.

## Disclosure

A.P. is a consultant for Genzyme Corporation and Shire Pharmaceuticals and has received investigator-initiated research support from Genzyme and Shire. These interests have been reviewed and managed by the University Federico II of Naples in adherence with its conflict of interest policies. The other authors declare no conflict of interest.
